# An assessment of the public health surveillance strategy based on molecular testing during three major pandemic waves of COVID-19 in Brazil

**DOI:** 10.1371/journal.pgph.0002164

**Published:** 2023-08-18

**Authors:** Lorena G. Barberia, Alexandra Boing, João Gusmão, Fabio Miyajima, Adriano Abud, Brigina Kemp, Marcela Zamudio, Tatiane C. Moraes de Sousa

**Affiliations:** 1 Department of Political Science, University of Sao Paulo, Avenida Professor Luciano Gualberto, Cidade Universitária, Sao Paulo, SP, Brazil; 2 Departamento de Saúde Pública, Programa de Pós-graduação em Saúde Coletiva, Universidade Federal de Santa Catarina, Campus Universitário Reitor João David Ferreira Lima, Centro de Ciências da Saúde, Trindade, Florianópolis, Santa Catarina, Brazil; 3 Multiprofessional Residency Program in Public Health Policy Management, School of Government, Fiocruz, Gleba A, Brasília, DF, Brazil; 4 Analytical Competence Molecular Epidemiology Laboratories, Genomic Surveillance Network, Oswaldo Cruz Foundation (FIOCRUZ), Ceara Branch–Eusebio, Rio de Janeiro, Brazil; 5 Faculty of Medicine, Postgraduate Programmes in Medical Sciences/Pharmacology/Pathology, Federal University of Ceara, Fortaleza, Brazil; 6 Instituto Adolfo Lutz, Prédio Central, Centro de Respostas Rápidas, Cerqueira Cesar, Sao Paulo, SP, Brazil; 7 Conselho de Secretários Municipais de Saúde—SP, Sao Paulo, SP, Brazil; 8 Department of Political Science, University of Sao Paulo, Cidade Universitária, Sao Paulo, SP, Brazil; Fundacao Oswaldo Cruz, BRAZIL

## Abstract

A national laboratory-based surveillance system was adapted to monitor the situation of SARS-CoV-2 in Brazil. The objective of the study was to compare the challenges in implementing COVID-19 surveillance strategies based on the Ministry of Health’s (MoH) distribution of RT-PCR tests to different types of laboratories. This retrospective study analyzed the MoH’s testing policies and distribution of RT-PCR tests to laboratories during the first, second, and third waves. Recipient laboratories were divided into groups: public health laboratories that belonged to the national network of public health laboratories (Group 1); public laboratories granted authorization during the pandemic (Group 2); and High-Capacity Testing Centers (Group 3). We analyzed the timing and duration of COVID-19 testing policies and the allocation of tests to laboratories by group and wave. Using t-tests, we analyzed the difference in the weekly average of tests distributed to labs by group and using Pearson’s correlation coefficient, analyzed the test distribution according to infection and death rates. Between epiweek 9, 2020, and epiweek 22, 2022, the MoH distributed an average of 263,004 RT-PCR tests per week. The weekly average of tests distributed was highest in the second wave (310,327 tests), followed by the first (218,005 tests) and third waves (201,226 tests). There was a significant increase in the mean weekly tests distributed in the second wave compared to the first and third waves (p = 0.047; IC 8.29–1110.71). We found a significant difference between the weekly average of tests distributed in the first and second wave (p < 0.001; IC -209.83–76.20) to Group 2. Group 3 received the second-highest number of tests from the MoH overall, with a reduction during the third wave to first-wave levels. The distribution of RT-PCR tests was not correlated with the case and death incidence.

## Introduction

Following the confirmation of the detection of the first cases of humans infected by Severe Acute Respiratory Syndrome Coronavirus 2 (SARS-CoV-2) in Wuhan, China [1], the publishing of the reference genome on 10 January 2020 enabled the introduction of the first molecular diagnostic tests to detect the virus RNA two weeks later [2]. By late January, the public health divisions of most countries were investing in surveillance testing to identify individuals possibly infected with SARS-CoV-2 [3,4]. These efforts were scaled up considerably in some countries as evidence quickly emerged that surveillance testing of suspected cases and contact tracing and isolation efforts were critical to containing the spread of the virus within communities and across countries [5–7]. As evidence from genomic epidemiology emerged that SARS-CoV-2 had moved beyond novel introductions to persistent local circulation with sustained transmission in most countries, public health authorities became further aware of the need to strengthen the monitoring of target regions within their jurisdiction where rising cases of infections were being reported [8].

However, even though diagnostic tests are essential for health surveillance and guiding government actions, there were significant gaps in the availability of supplies and the existing structure of laboratory networks which negatively impacted the processing capacity of molecular tests and delayed the issuing of diagnostic reports in many countries. These challenges are widely recognized to have compromised the effectiveness of the early pandemic response [9–11]. Furthermore, there are vast inequities in access to testing across and within countries. According to WHO ACT-Accelerator, only 0.4% of global COVID-19 tests were performed in low-income countries [12].

In Brazil, unlike most developing countries [11], the nation’s comprehensive and universal public health system includes a network of public health laboratories divided into national, regional, state, city, local, and border levels. This network was established decades before the arrival of COVID-19. Its core structure centers on relatively large, medium to high-throughput, centralized laboratories with semi-automated equipment and trained personnel capable of analyzing and processing samples and providing diagnostic services in the public health system. Also, it includes research centers linked to the public health system in each state of the Brazilian federation [13]. Notwithstanding these resources, since the onset of the pandemic in 2020, Brazil has faced many challenges which limited its capacity to conduct testing with real-time reverse transcription polymerase chain reaction (RT-PCR) tests for SARS-CoV-2.

This article analyzes the Ministry of Health’s (MoH) distribution of RT-PCR tests to laboratories within the SUS over the first three pandemic waves between February 2020 and June 2022. We describe the efforts directed at expanding the public health laboratory network in the early stage of the first wave and assess how resource allocation decisions panned out as the spread of SARS-CoV-2 continued to exert a heavy toll on SUS during the second and third waves.

## Background

Molecular testing capabilities affect the ability to contain, mitigate, and clinically manage infectious diseases. When laboratory surveillance and processing capacity in the public health sector are modest with limited resources for improvements, there are substantial risks that critical delays in obtaining reliable indicators over the emergence and spread of an epidemic threat, potentially masking the actual case burden will occur. These lags prevent timely awareness, thus slowing the introduction of appropriate clinical and epidemiological policies for a public health emergency response.

The National System of Public Health Laboratories (SISLAB), which includes the National Network of Epidemiological Surveillance Laboratories, is a fundamental pillar of the Brazilian unified public health system (SUS). SISLAB comprises national networks of epidemiological surveillance, environmental health, sanitary, and high-complexity medical assistance. These networks are organized into subnetworks composed mainly of collaborating and reference laboratories at different levels: national, regional, state, municipal, local, and frontier. The network includes state reference laboratories, which are also called public health central laboratories (LACEN), that are in all 27 Federation Units (UF) (26 States and the Federal District). The regional and national reference laboratories that are recognized by the World Health Organization (WHO) are the Laboratory of Respiratory Viruses and Measles at Instituto Oswaldo Cruz at Oswaldo Cruz Foundation (Fiocruz), located in Rio de Janeiro state; the Laboratory of Respiratory Viruses at Instituto Adolfo Lutz in Sao Paulo state (IAL/SP), and Instituto Evandro Chagas in Para state (IEC/SVS/MS). In addition to these international reference laboratories, SISLAB has an organized network of reference laboratories at the subnational levels. Before the pandemic, the laboratories that were part of the SISLAB public network included the LACENs, federal reference laboratories (including those within FIOCRUZ), public health laboratories (such as those run by local authorities), and other MoH collaborators.

In early 2020, the MoH’s early response to COVID-19 centered on the training and certification of the molecular diagnosis of COVID-19 and the launching of COVID-19 High-Capacity Testing Centers (HTC). Testing certification was completed for the three WHO national reference laboratories on 31 January 2020 and 18 March 2020 for the twenty-seven LACENs. COVID-19 HTCs were established at Fiocruz in Rio de Janeiro, at the Institute of Molecular Biology in Parana state, and at Fiocruz in Ceara. Additionally, a public-private partnership of a high-testing platform was established with Diagnostica da America S.A.–DASA, one of Latin America’s largest private diagnostic laboratories. In addition, several laboratories from public institutions were certified into the MoH laboratory network, such as public laboratories in the agriculture, security, defense, and university sectors.

## Methods

### Data sources

The key policies and the number of COVID-19 RT-PCR tests distributed according to the receiving institution or laboratory from each of Brazil’s federation units (states and the federal district) were obtained from official documents released by the MoH and from weekly epidemiological COVID-19 bulletins [14–17]. Data regarding the number of COVID-19 cases and deaths were also collected from the COVID-19 dashboard maintained by the MoH [18]. The number of inhabitants per federation unit, used to estimate the incidence of COVID-19 cases per 100,000 inhabitants, was obtained from the Brazilian Institute of Geography and Statistics (IBGE) [19]. The data can be accessed from the following repository: https://github.com/cgrtbrfed/covid19brpolicyresponses.

### Stage 1: Laboratory classification

The laboratories listed by the MoH COVID-19 weekly epidemiological bulletins as recipients of RT-PCR tests were classified into three groups based on two criteria. First, laboratories were categorized into Group 1 if they were part of the National System of Public Health Laboratories (SISLAB) and, therefore, already operating as part of the national surveillance system for mandatory notifiable diseases. We assessed whether the remaining recipient laboratories were public laboratories not previously linked to the MoH, such as municipal or state health departments (Group 2), or initiatives created by the MoH during the pandemic (Group 3). We then estimated the weekly average of tests allocated by the MoH to each laboratory over the first three waves in Brazil.

### Stage 2: Mapping the type and duration of MoH SARS-CoV-2 testing policies

Based on an analysis of MoH documents, we identified the dates and duration of the key policies adopted by the federal government regarding SARS-CoV-2 testing in Brazil from the beginning of 2020 to June 2022. In addition to RT-PCR testing policies, we also identified serological and antigen testing policies. Each policy’s intervention duration was calculated from when measures were announced until the end date or the investigated study period.

Each epidemiological wave was defined from the period of exponential expansion to the marked decline in COVID-19 cases in Brazil between March 2020 and June 2022. The first wave occurred from epi week 9 to 43 of 2020 (35 weeks). The second wave lasted approximately 62 weeks (between epi week 44 of 2020 and 52 of 2021); the third wave was between epi weeks 1 and 22 of 2022 (22 weeks). The waves are similar to studies examining differences across pandemic waves in Brazil’s COVID-19 case incidence, lethality, and vaccination [20,21].

### Stage 3: Statistical analysis

Based on the laboratory categories described in Table 1, using a Student’s t-test (t student), we analyzed the difference in the weekly average of tests distributed to labs in the preexisting SISLAB network (Group 1) and the public labs not previously linked to MoH (Group 2). In this and all other statistical analyses, significant results were considered for p < 0.05.

**Table 1:** Description of recipient lab categories between 16 February 2020 and 25 June 2022 in Brazil.

CATEGORY	DESCRIPTION
***Public health laboratories certified by the MoH as part of SISLAB before the COVID-19 pandemic (Group 1)***	
Public Health Central Laboratories (LACENs)	Central laboratories are localized in each federation unit’s capital city and connected to the MoH’s general coordination of public health laboratories (CGLAB).
Reference Laboratories	Public laboratories certified by the MoH for the diagnostic testing of selected pathogens (including respiratory viruses). The WHO recognizes reference labs with national status.
Collaborative Laboratories	Public health collaborating institutions of the MoH associated with the Ministry of Justice
Public Health Laboratories	Laboratories associated with public healthcare units from the state or local authorities
***Public Laboratories certified by the MoH but not previously part of SISLAB before the pandemic (Group 2)***	
Contracted Laboratories	Analytical laboratories contracted by public health authorities to conduct diagnostic testing.
University Laboratories	University-based laboratories from the public health sector (federal and state)
Other Public Laboratories	Public laboratories outside the health sector, e.g., agriculture and biotechnology research laboratories
***Covid-19 High-Capacity Testing Centers (HTCs) created by the MoH (Group 3)***	
Rio de Janeiro (RJ) HTC	COVID-19 Diagnostic Support Unit located at Fiocruz Rio de Janeiro (UNADIG-RJ)
Ceara (CE) HTC	COVID-19 Diagnostic Support Unit located at Fiocruz Ceara (UNADIG-CE)
Parana (PR)/IBMP HTC	COVID-19 Diagnostic Support Unit located at the Institute of Molecular Biology of Parana (IBMP), Fiocruz PR
Sao Paulo (SP)/DASA HTC	A private-owned laboratory (DASA) was awarded a contract to handle exams referred by the COVID-19 Emergency Diagnostic Center from Sao Paulo state.

To understand the extent to which the intensity of COVID-19 RT-PCR tests in the FUs in the second and third epidemiological waves was driven by test distribution and case and death incidence in prior waves, we estimated Pearson’s correlation coefficients. The correlation coefficient (*r*) ranges between -1 and 1, with values closer to zero, indicating a weaker correlation. For each laboratory, lab group, and each state, we evaluated the correlation coefficients using the weekly average of tests distributed by laboratory category in the second and third waves and the incidence of COVID-19 cases and deaths per 100,000 inhabitants registered in the previous wave (first and second wave, respectively). The tests distributed to HTCs were not included in this analysis as these centers received tests to process from public health units across the federation and not strictly in the state where these facilities were located.

## Results

### MoH COVID-19 testing policies

**Fig 1:**
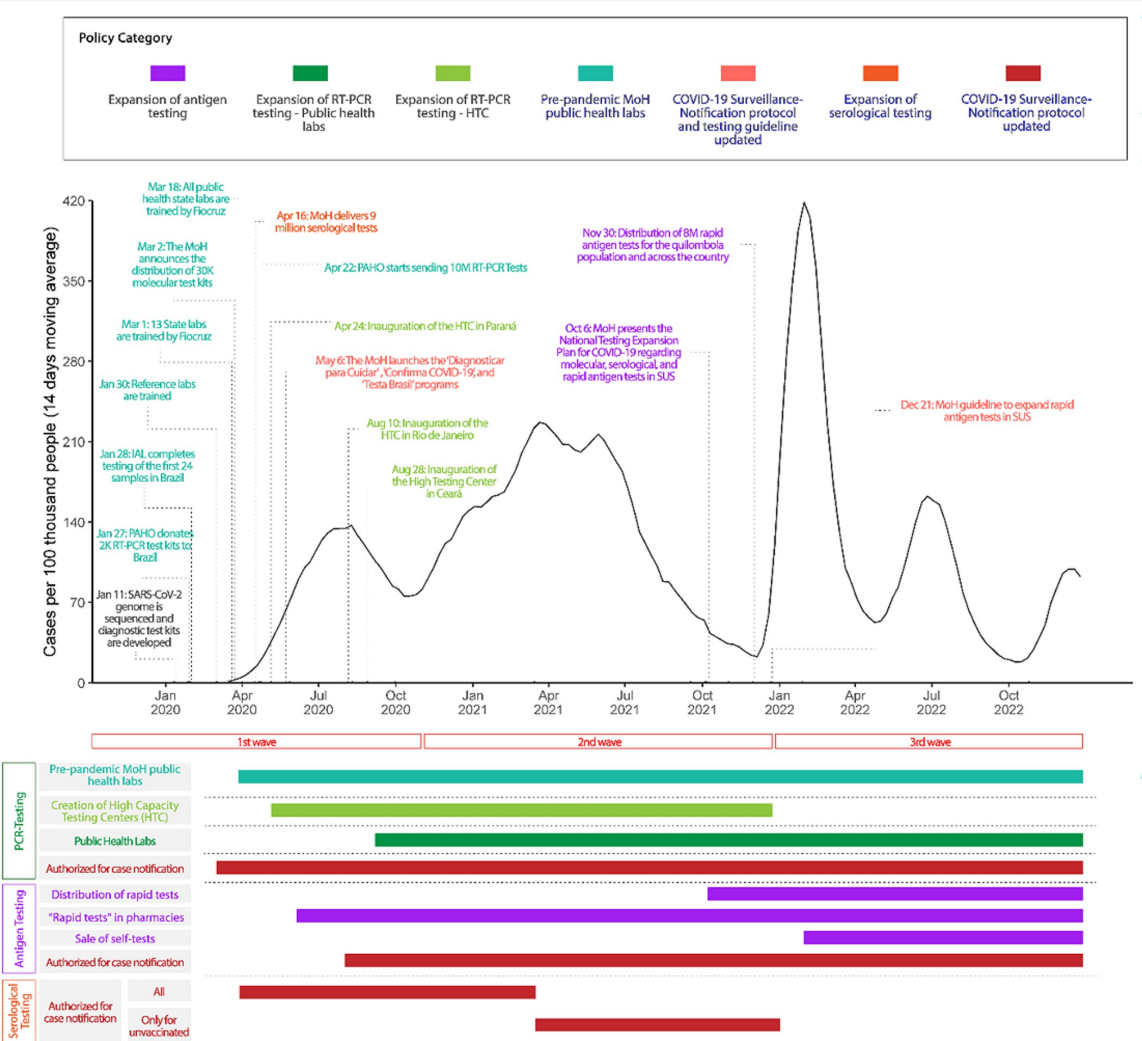
Timeline of Brazil’s MoH’s COVID-19 testing policies.

Fig 1 depicts the duration of testing policies juxtaposed with the evolution of cases per 100,000 during the three major epidemic waves. The majority of MoH policies that affected COVID-19 diagnosis and surveillance, including molecular (RT-PCR), serological, and antigen testing, were concentrated in the first epidemic wave. The MoH published the first version of the COVID-19 notification guidelines in the initial stage of the first wave (April 2020), while a second version was issued in August 2020 [22,23]. The first guideline permitted cases to be confirmed by either serological or molecular-based tests or clinical-epidemiological criteria. This policy remained in effect until the revised third version of the guideline was introduced in March 2021, when serological test notification was finally suppressed as a diagnostic criterion amid the second severe pandemic wave [24]. Although results from rapid antigen tests were accepted as a notification criterion since August 2020, it only became more predominant as a COVID-19 testing tool during the second and third waves [25,26] (S1 Table).

The policies introduced in the first wave of COVID-19 referred to the training and qualification of molecular testing in public health laboratories that were part of the SISLAB (Group 1), the easing requirements to enable public laboratories to conduct molecular testing for COVID-19 (Group 2) and the establishment of HTCs (Group 3). In addition, the MoH invested efforts in expanding the distribution and using serological tests to aid COVID-19 diagnostics. During the second and third waves, most policy interventions focused on regulating the use and distribution of rapid antigen tests.

**Fig 2:**
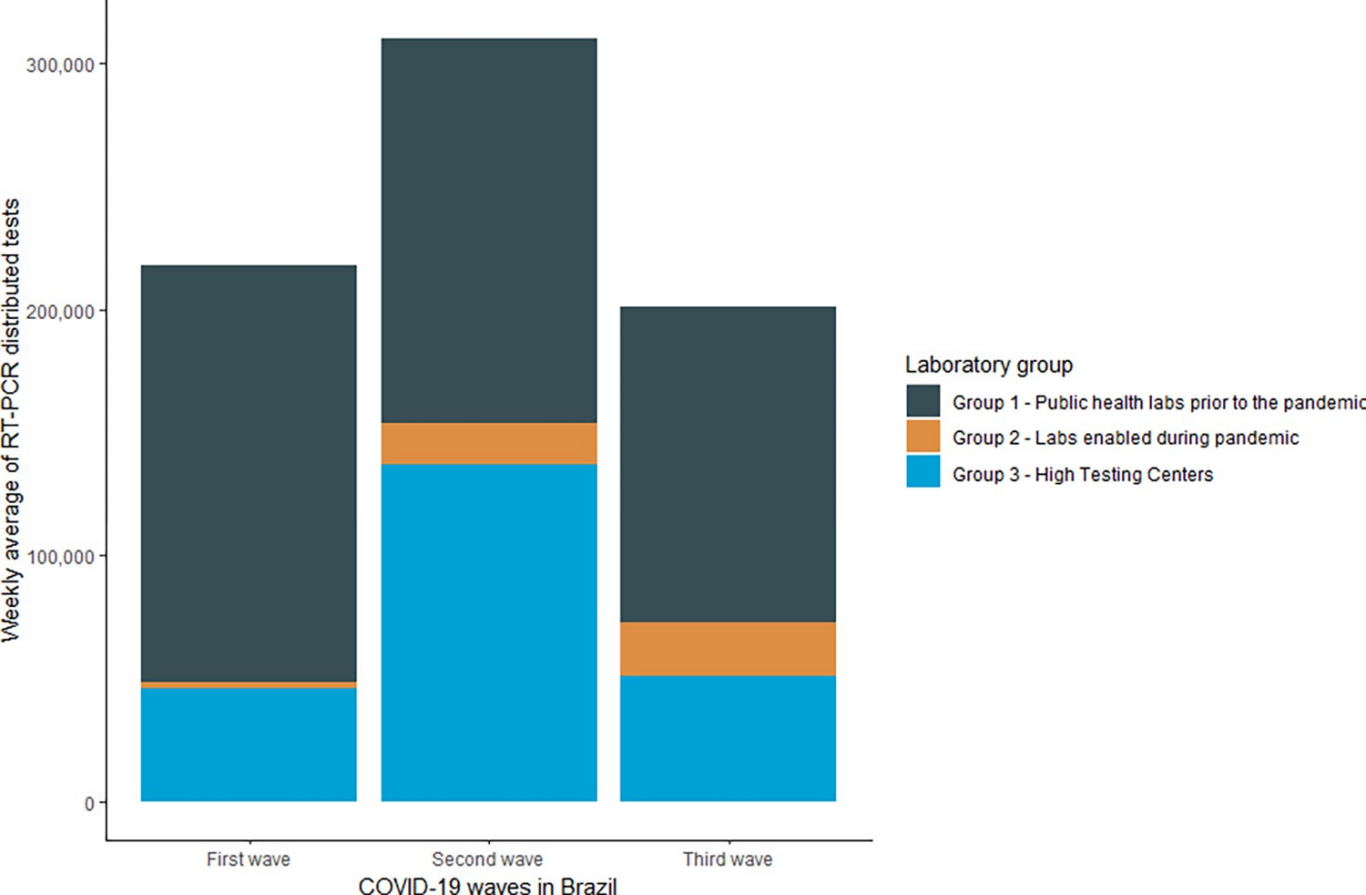
Weekly average RT-PCR tests distributed to Group 1, 2, and 3 labs during the first, second and third COVID-19 waves in Brazil, 2020–2022.

Table 1 reports the description of the COVID-19 molecular laboratories which were classified as public health laboratories certified by the MoH as part of SISLAB before the COVID-19 pandemic (Group 1), public laboratories certified by the MoH but not previously part of SISLAB before the pandemic (Group 2) and Covid-19 High-Capacity Testing Centers (HTCs) created by the MoH (Group 3). Considering these three groups 31,297,432 RT-PCR tests were distributed between epiweek 9, 2020, and epiweek 25, 2022, with a weekly average of 263,004. The weekly average of tests distributed was highest in the second wave (310,327 tests), followed by the first (218,005 tests) and third waves (201,226 tests) (Fig 2). The number of labs receiving tests was also highest in the second wave (170). During the first and third waves, we identified 67 and 64 laboratories in receipt of such tests, respectively.

#### First wave

The first epidemiological wave of COVID-19 started on 23 February 2020 (epiweek 9) and ended on 24 October 2020 (epiweek 43). In this first wave, the laboratory network was gradually scaled up. Although the SARS-CoV-2 virus was sequenced on 7 January 2020, Brazil only received the RT-PCR test kits in the last week of January when PAHO donated two thousand RT-PCR test kits on 27 January 2020 [17]. These first tests were used to develop laboratory testing capabilities in the three WHO reference laboratories in Brazil: Fiocruz (Rio de Janeiro State), Instituto Adolfo Lutz (Sao Paulo State), and Instituto Evandro Chagas (Para State). MoH reports state that the total testing capacity for SARS-CoV-2 was 584 RT-PCR tests in the public health laboratory network in February 2020. By March 2020, the MoH distributed 45,240 RT-PCR tests for epidemiologic surveillance efforts. The majority of these tests were manufactured locally by Fiocruz. However, since local transmission was confirmed on 20 March 2020 [27], the demand for RT-PCR testing capabilities was greater than existing capabilities in most states when tests arrived in these localities. In the 35-week period, the MoH distributed 7,630,176 RT-PCR tests, representing 24.4% of the total tests distributed over the three waves, with a weekly average of 218,005.

### Public health SISLAB laboratories

**Fig 3:**
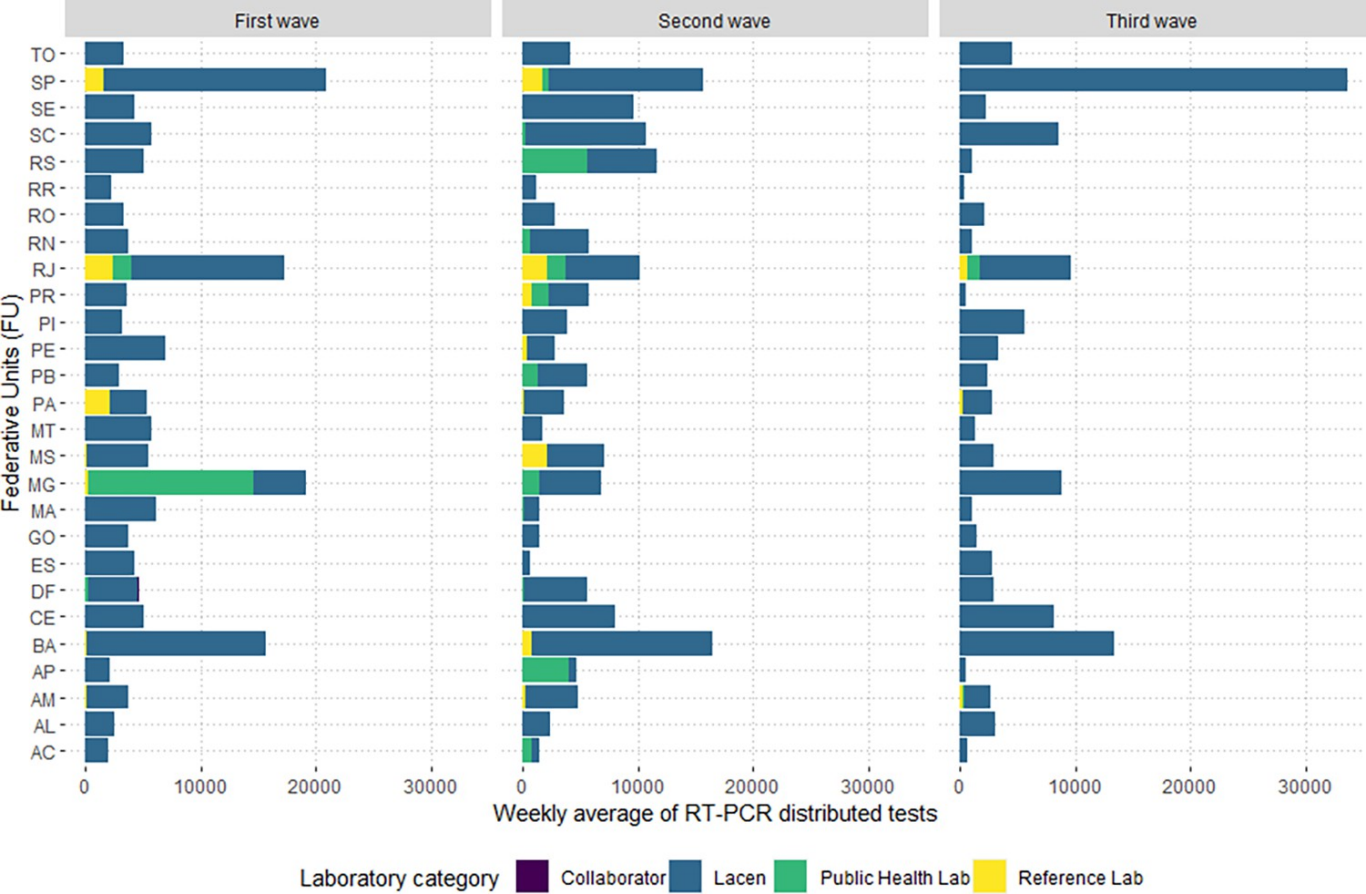
Weekly average of distributed RT-PCR tests received by Group 1 labs by state during the first, second, and third COVID-19 waves in Brazil, 2020–2022.

**Fig 4:**
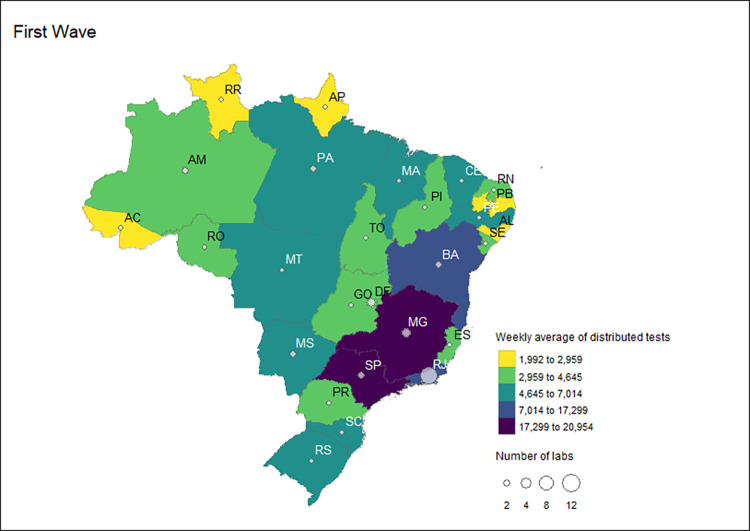
Number and distribution of Group 1 labs and the weekly average of distributed RT-PCR tests received by these laboratories by state during the first COVID-19 wave in Brazil, 2020–2022.

Note: The maps were created using the R software program with the "geobr" [28] and "tmap" [29] packages. The shapefiles used for data visualization were provided by the "geobr" package [28].

Group 1 laboratories, already in operation before the pandemic (SISLAB), received 77.7% of tests in the first wave, with a weekly average of 169,395 (n = 5,928,816). All FUs received tests for this category of laboratories, mainly due to the presence of LACEN in all FUs (Fig 3). However, the states with the highest number of laboratories linked to SISLAB before the pandemic were Rio de Janeiro (11 labs), and Distrito Federal and Minas Gerais, with three labs each (Fig 4). The FUs of Sao Paulo (20,954 tests), Minas Gerais (19,205), Rio de Janeiro (17,299) and Bahia (15,695) received the highest overall volume (Fig 4). All other FUs received less than 10,000 tests per epidemiological week in the first wave.

### Public laboratories certified by the MoH during the pandemic

**Fig 5:**
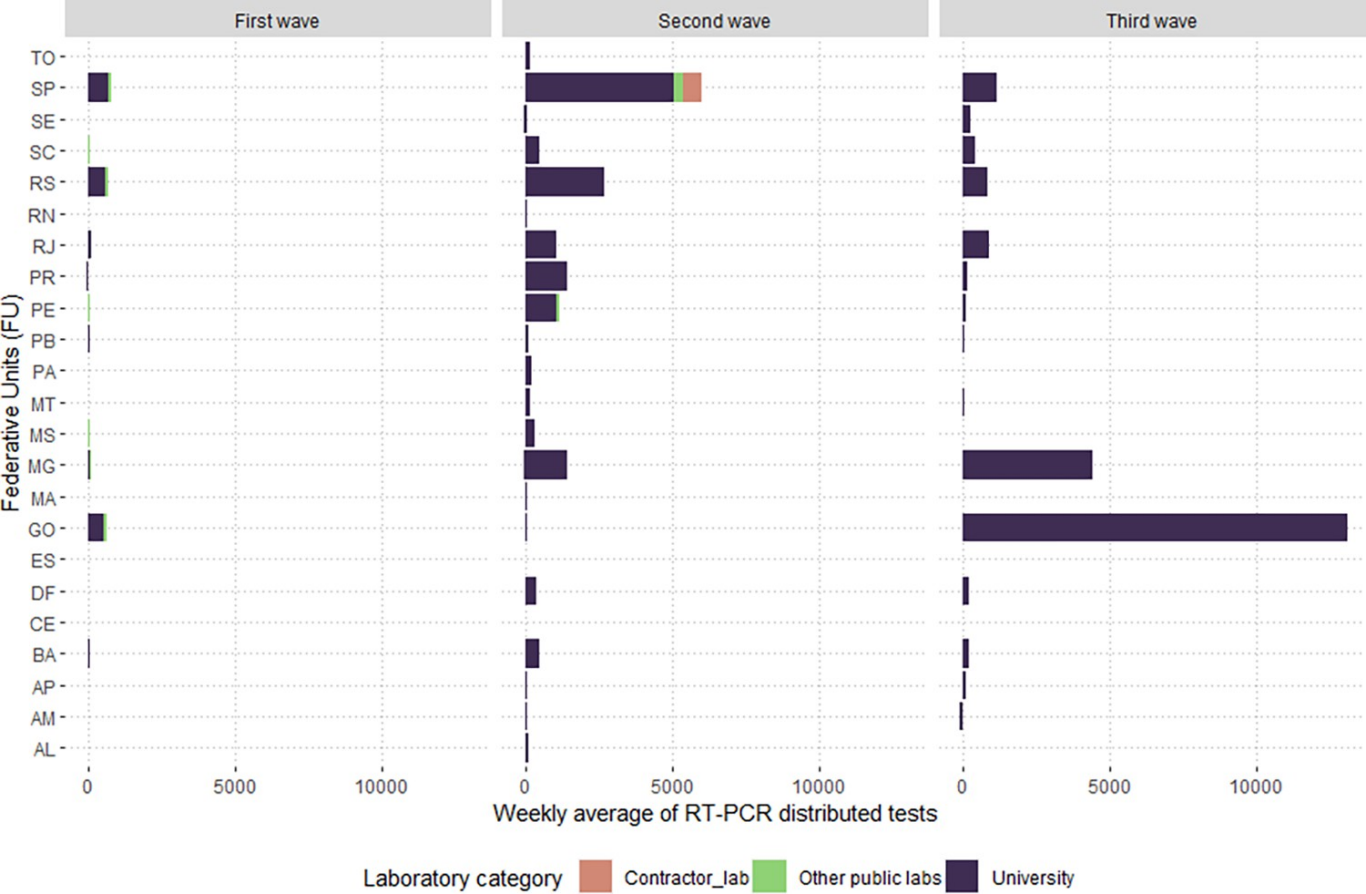
Weekly average of distributed RT-PCR tests received by Group 2 labs by state during the first, second, and third COVID-19 waves in Brazil, 2020–2022.

**Fig 6:**
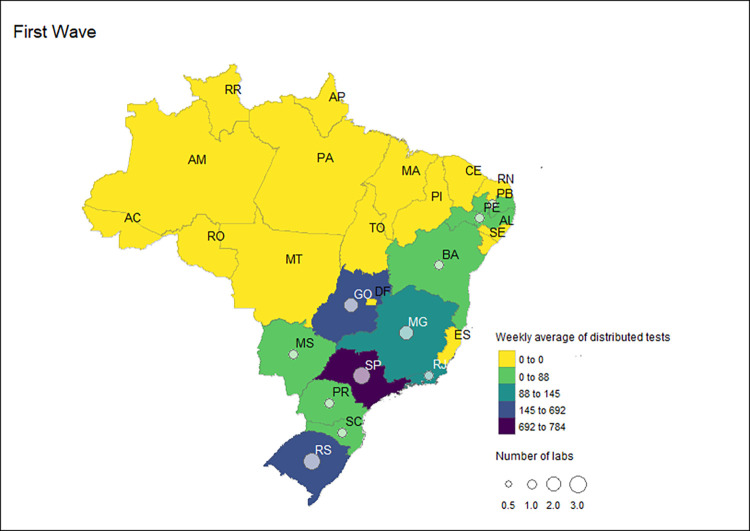
Number and distribution of Group 2 labs and the weekly average of distributed RT-PCR tests received by these laboratories by state during the first COVID-19 wave in Brazil, 2020–2022.

Notes: The maps were created using the R software program with the "geobr" [28] and "tmap" [29] packages. The shapefiles used for data visualization were provided by the "geobr" package [28].

During the first wave, 17 Group 2 labs received COVID-19 RT-PCR tests. Of these laboratories, ten were associated with universities, and seven were other types of public laboratories, as classified in Table 1. Group 2 laboratories received 1.3% of the tests distributed in the first wave, with the lowest value recorded for this group of laboratories in the three waves of COVID-19 (Fig 5). In the first wave, we identified a weekly average of 2,545 tests and 99,568 tests in the 35-week period (Fig 6).

There was a greater concentration of Group 2 laboratories in states in the south and southeast regions. These laboratories were distributed in 11 states: Sao Paulo and Rio Grande do Sul, with three laboratories, Goias and Minas Gerais, with two laboratories, and Bahia, Mato Grosso do Sul, Paraiba, Pernambuco, Parana, Rio de Janeiro and Santa Catarina with one laboratory (Fig 6). Sao Paulo received the highest weekly average of tests in the first wave, with an average of 784 tests per week. Rio Grande do Sul and Goias, with a weekly average of 692 and 647 tests, respectively (Fig 6), were the second and third-ranking states regarding the volume of tests.

### Covid-19 High-Capacity Testing Centers (HTCs)

In April 2020, the MOH announced it would expand its testing capacity by establishing HTCs [30]. Four HTCs were launched in the first wave (Fig 1). According to the MoH [22], these facilities began processing tests in the second half of 2020 in the states of Ceara, Parana and Rio de Janeiro managed and operated by the Oswaldo Cruz Foundation (FIOCRUZ), a national institute of health of the MoH with branches in these federal units. The fourth HTC was located in the state of Sao Paulo and was managed in partnership with a private laboratory group (DASA). These HTCs were introduced to not only receive samples from the states where they were located, but also to meet the needs of other states, in accordance to the MoH’s general coordination of central public health laboratories (CGLAB) [21].

**Fig 7:**
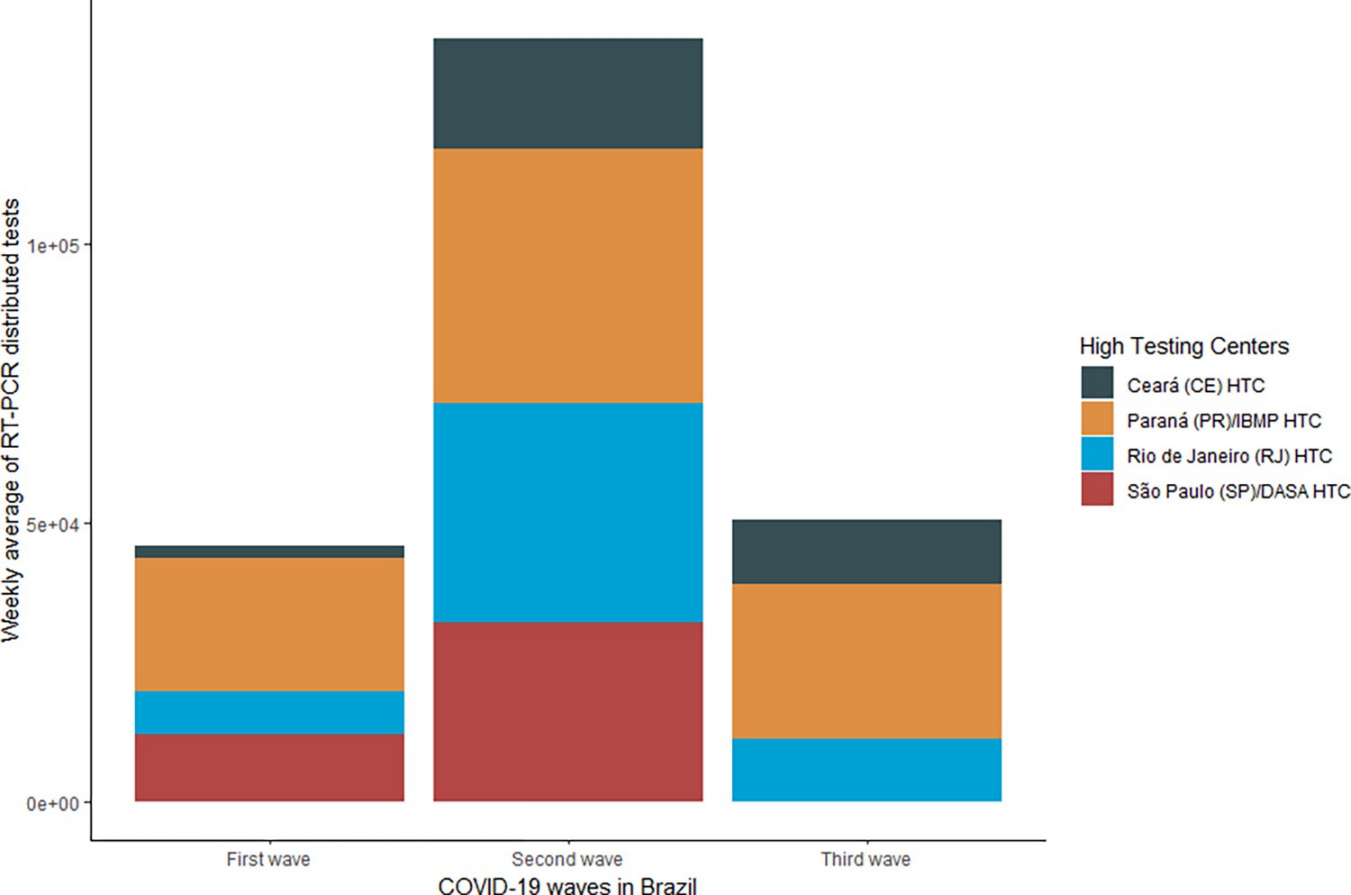
Weekly average RT-PCR tests distributed to Group 3 labs during the first, second and third COVID-19 waves in Brazil, 2020–2022.

All four HTCs in Group 3 received COVID-19 RT-PCR tests during the first wave. A total of 1,601,792 tests were distributed to these labs (14,3% of the share of tests deployed in the first wave). The Parana HTC received 52.5% of these tests (n = 840,192), the Sao Paulo/DASA HTC received 26.2% (n = 419,936), and the Rio de Janeiro HTC 16.9% (n = 270,240 tests). The Ceara HTC received 4.5% (n = 71,424 tests) of the distributed RT-PCR tests in this wave (Fig 7).

#### Second wave

The second wave comprises 62 weeks from epiweek 44 of 2020 to 52 of 2021 (25 October 2020 and 1 January 2022). During this wave, 19,240,280 COVID-19 RT-PCR tests were distributed by the MoH, representing 61.5% of the total of distributed tests until epiweek 25 of 2022, with a weekly average of 310,327 tests.

### Public health SISLAB laboratories

**Fig 8:**
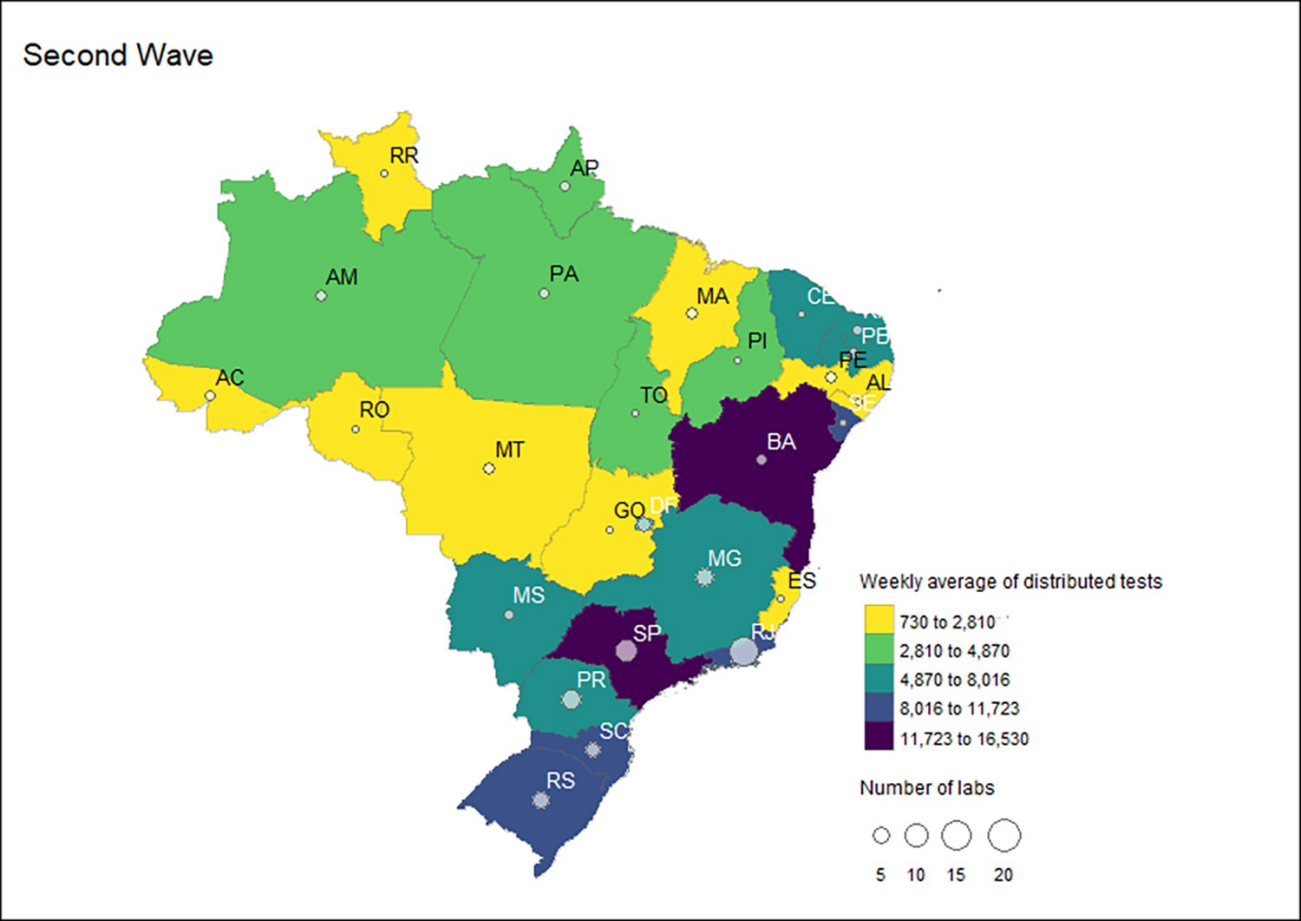
Number and distribution of Group 1 labs and the weekly average of distributed RT-PCR tests received by these laboratories by state during the second COVID-19 wave in Brazil, 2020–2022.

Group 1 laboratories received 9,714,864 COVID-19 RT-PCR tests and a weekly average of 156,691 tests. These represented 50.5% of the total tests distributed by the MoH in the second wave. Of this total, 8,069,620 were distributed to the LACEN labs, 1,132,876 tests were to other public health labs, 511,068 were sent to reference labs, and 1,300 tests were to collaborator labs. Thus, most tests were sent to LACENs (83.1%) and other public health labs (11.7%). In the second wave, 86 laboratories in Group 1 received MoH tests. Of these, 31 were LACEN, 15 were reference laboratories, 38 were public health laboratories, and two were collaborating laboratories. Rio de Janeiro had the highest number of Group 1 laboratories, mainly due to the presence of health units with public health laboratories (Fig 8). Bahia, with an average of 16,530 tests and Sao Paulo, with a weekly average of 15,703 tests received the first and second highest number of tests (Fig 8).

### Public laboratories certified by the MoH during the pandemic

**Fig 9:**
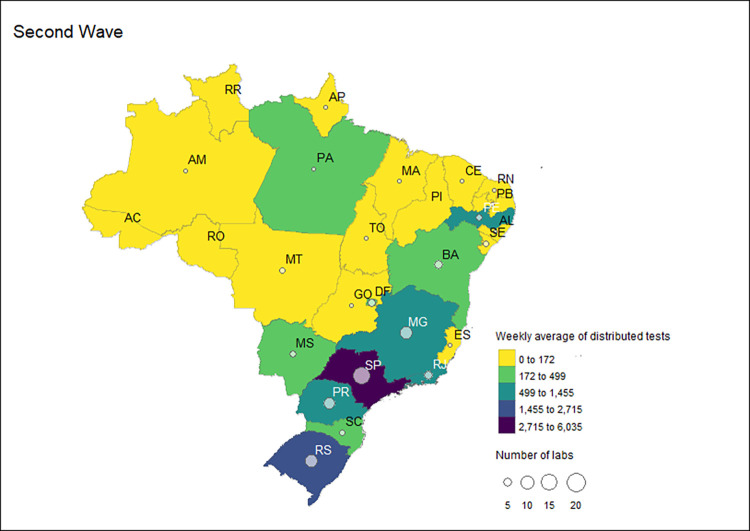
Number and distribution of Group 2 labs and the weekly average of distributed RT-PCR tests received by these laboratories by state during the second COVID-19 waves in Brazil, 2020–2022.

Group 2 laboratories received 5.4% (1,036,508 tests) of the distributed RT-PCR tests in the second wave. During this period, the MoH increased receiving laboratories from 17 in the first wave to 80. Of this total, the MoH concentrated its strategy on sending tests to (88.8% or n = 71) university labs. Tests were also sent to contractor labs (n = 7), and public laboratories in the non-health sectors (n = 2). The highest proportion of RT-PCR tests distributed in Group 2 were for laboratories linked to universities (93.4%; 967,908 tests), contracted laboratories (4.1%; 42,600 tests), and other public laboratories outside the health sector (2.5%; 26,000 tests). This group’s highest laboratories were in Sao Paulo, with 17 receiving laboratories in Group 2. The other states were Minas Gerais and Rio Grande do Sul, with nine laboratories each, and Parana, with eight laboratories (Fig 9).

Five states received the highest weekly average of tests during the second wave in Group 2: Sao Paulo (6,035 tests), Rio Grande do Sul (2,715 tests), Minas Gerais (1,455 tests), Parana (1,450), Pernambuco (1,172), and Rio de Janeiro (1,075). On average, the labs in all other states received less than 1,000 tests per week in the second wave, as shown in Fig 9.

### Covid-19 High-Capacity Testing Centers (HTCs)

The HTCs were mainly used during the second wave (Fig 2). In this wave, the MoH distributed 8,488,908 COVID-19 RT-PCR tests (44.1% of tests) to these centers. The HTC located in Parana/IBMP received 2,826,704 RT-PCR tests (33,3%), the Rio de Janeiro HTC received 2,430,496 tests (26.6%), the Sao Paulo/DASA received 1,996,840 (23.5%), and the Ceara HTC received 1,234,868 (14.5%) (Fig 7).

There is a weak negative correlation without statistical significance between the mean weekly tests received by Group 2 laboratories per state in the second-wave and first-wave cases (r = -0.34; p = 0.083) (S1 Fig) and deaths (r = -0.15; p = 0.46) (S2 Fig). In addition, a negative correlation is verified in the allocation of RT-PCR tests to Group 1 laboratories in the second-wave and COVID-19 first-wave cases (r = -0.33; p = 0.092). The correlation between second-wave testing allocation and deaths in the first-wave is also not statistically significant for Group 1 (r = -0.17; p = 0.39).

#### Third wave

The third wave period analyzed in this study occurred between epiweeks 1 to 22 of 2022, which is the period with the highest number of cases in Brazil following the arrival and spread of the Omicron variants. Although consisting of only 22 weeks, this is the wave in which the MoH distributed the lowest number of RT-PCR tests. During this period, 4,426,976 COVID-19 RT-PCR tests were distributed by the Brazilian MoH, representing 14.1% of the total number of tests. However, the number of tests is similar to the tests distributed in the first wave on an average weekly basis (Fig 2).

### Public health SISLAB laboratories

During the third wave, the MoH allocated most tests to group 1 laboratories which received 2,824,832 COVID-19 RT-PCR tests (63.8% of the total tests received during the third wave). Similar to the preceding waves, these tests were distributed primarily to the 28 LACEN units (98.1%). All LACENs received RT-PCR tests; however, the state of Santa Catarina had two LACEN units receiving tests in the third wave, totaling 28 LACEN units. Six Group 1 reference labs in four states received 1% of tests. Of these, only Rio de Janeiro had more than one reference laboratory (n = 3) that received tests during this period. The other states with reference laboratories receiving tests in the third wave were Amazonas, Minas Gerais and Para.

**Fig 10:**
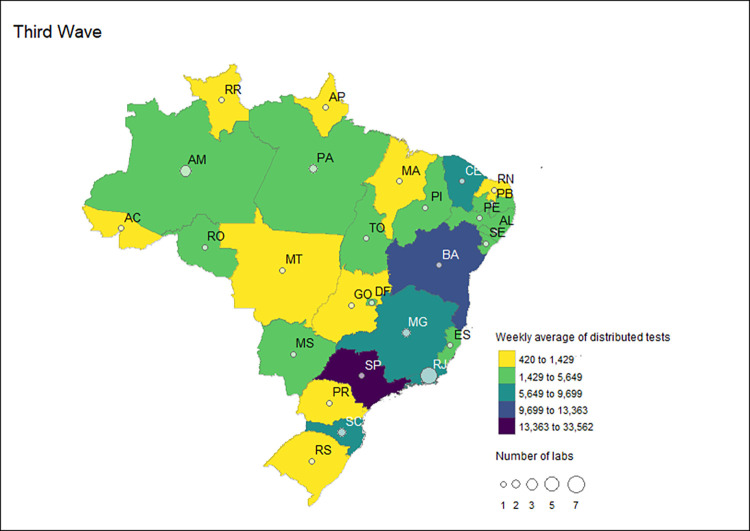
Number and distribution of Group 1 labs and the weekly average of distributed RT-PCR tests received by these laboratories by state during the third wave in Brazil, 2020–2022.

Overall, the states with the highest number of laboratories in this category were Rio de Janeiro, with seven units; Amazonas, with three units; and Minas Gerais, Para and Santa Catarina, with two units each. The states with the highest weekly average of tests received in the third wave were Sao Paulo (33,562), followed by Bahia (13,363). The laboratories in the remaining states received less than 10,000 tests on average per week in the third wave (Fig 10).

Considering the distribution of tests stratified by federal units of Group 1, the total number of laboratories receiving the distributed diagnostic tests and the weekly average varied across the pandemic waves. The states of Sao Paulo, Bahia, Rio de Janeiro, Minas Gerais, and Ceara were identified as the states that were allocated the highest weekly average of tests over the study period. Rio de Janeiro had the highest number of laboratories in receipt of molecular tests for all waves analyzed. The MOH surveillance strategy relied exclusively upon Group 1 labs in four Brazilian FU in the three waves: Acre, Piaui, Roraima, and Rondonia.

### Public laboratories certified by the MoH during the pandemic

**Fig 11:**
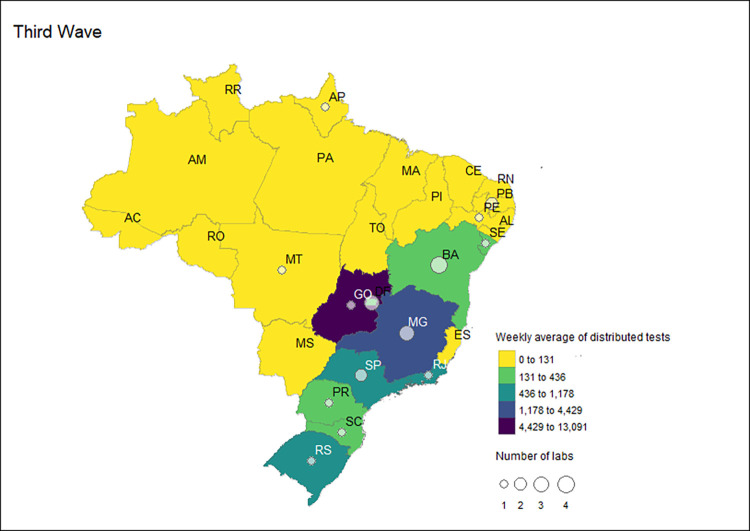
Number and distribution of Group 2 labs and the weekly average of distributed RT-PCR tests received by these laboratories by state during the third wave in Brazil, 2020–2022.

There was a considerable drop in newly certified public laboratories integrated into the MoH COVID-19 network during the COVID-19 pandemic that received tests in the third wave. While 80 public laboratories received tests in the second wave, in the third wave, only 23 of these laboratories received molecular tests and were all university laboratories. The laboratories of the universities that received the MoH tests were located in 14 states, with the largest number of universities in Bahia (n = 4), the Federal District and Minas Gerais (n = 3), and in the states of Paraiba and Sao Paulo, with two labs each. In the other nine states (Amapa, Goias, Mato Grosso, Pernambuco, Parana, Rio de Janeiro, Rio Grande do Sul, Santa Catarina and Sergipe), only one university laboratory received MoH tests in the third wave (Fig 11).

Although the number of Group 2 laboratories was lower in the third wave, these labs received the highest number of tests among all waves, with a total of 488,064 of RT-PCR tests sent in this period. Regarding the average volume of tests received, there was an increase from an average of 16,718 tests per week in the second wave to an average of 22,184 tests per week. Goias received the highest weekly average of RT-PCR tests (13,091 tests) in the third wave, followed by the states of Minas Gerais (4,429) and Sao Paulo (1,178). In other states, Group 2 laboratories received, on average, less than 1,000 tests per week in the third wave (Fig 11).

### Covid-19 High-Capacity Testing Centers (HTCs)

Only three HTCs received tests in the third wave (Parana/IBMP HTC, Ceara HTC, and Rio de Janeiro HTC). The total number of tests distributed to these units in this period was 1,114,080, representing 25.2% of the tests distributed in the third wave. The Parana unit received 55.2% of the tests, with a weekly average of 27,971 tests, the Ceara unit received 22.8%, a weekly average of 11.542 tests, and the Rio de Janeiro unit received 22% of the tests, a weekly average of 11,127 tests (Fig 7).

No statistical evidence confirms that the MoH distributed more tests to laboratories in states in the third wave considering cases and deaths per 100 thousand inhabitants registered in the second wave. There is a weak positive correlation without statistical significance (r = 0.098; p = 0.63) between the mean number of RT-PCR tests distributed per week for each state in the third wave and the registered cases in the second wave for Group 2 laboratories, whereas there is a negative correlation without statistical significance (r = -0.15; p = 0.45) for Group 1 laboratories (S3 Fig). Considering the COVID-19 deaths per 100 thousand inhabitants, there are positive correlations without statistical significance between the weekly average of tests distributed in the third week and the deaths in the second wave for Group 1 labs (r = 0.11; 0.59) and Group 2 (r = 0.26; p = 0.2) (S4 Fig).

With more than 10 million tests received (n = 11,204,780), the weekly average of HTCs (Group 3) was 94,158 tests. The HTCs received the second highest amount of tests from the MoH over the investigated period, corresponding to 35.8% of the overall number of RT-PCR tests. The number of tests received by these Group 3 labs varied greatly over the three epidemiological waves, with the highest weekly average of tests received in the second wave (136,918 tests per week), followed by the third wave (50,640 tests) and the first wave (45,765). Over the period studied, the unit that received the highest average of tests per epidemiological week was the Parana/IBMP-Fiocruz/PR HTC unit (35,985 tests), followed by the HTC unit in Rio de Janeiro (24,752 tests), Sao Paulo/DASA (20,309 tests), and the Ceara HTC (13,111 tests). According to the epidemiological bulletins published by the MoH, the HTCs located in Parana, Rio de Janeiro, and Ceara received tests in the three analyzed waves. In contrast, the Sao Paulo/DASA HTC received tests in the first and second waves (Fig 7).

#### Comparison of molecular testing allocation across the pandemic waves

Table 2 reports the results of tests comparing weekly average tests distribution in the three pandemic waves overall, and for laboratory groups 1 and 2. A comparison was not performed for group 3 labs since there were only four labs in this category. Overall, the drop in the mean of weekly tests distributed to each laboratory in the third wave was statistically different from zero (p = 0.047).

**Table 2:** Student’s t-test result of the difference of means tests of weekly average test receipt during the first, second and third waves in Brazil, 2020–2022.

	95% Confidence Interval (CI)	T-Test	DF	*P-value*
**Overall**				
**First wave x Second wave**	-994.04; 47.15	-1.7936	194	0.074
**First wave x Third wave**	-245.71; 417.8	0.5115	194	0.610
**Second wave x Third wave**	8.28; 1110.7	2.0019	194	0.047*
**Group 1—Public health lab linked to the Ministry of Health before COVID-19**				
**First wave x Second wave**	-330.31; 600.59	0.5765	93	0.566
**First wave x Third wave**	-83.31; 955.51	1.6673	93	0.099
**Second wave x Third wave**	-219.45; 821.37	1.1484	93	0.254
**Group 2—Public Labs not previously linked to the Ministry of Health**				
**First wave x Second wave**	-209.83; -76.21	-4.2491	96	0.000**
**First wave x Third wave**	-483.26; 84.5	-1.3941	96	0.166
**Second wave x Third wave**	-347.75; 235.03	-0.384	96	0.702

* p<0.05

** p<0.01.

### Public health SISLAB and public labs certified by MoH during the pandemic

Group 1 laboratories received nearly 20 million tests (n = 18,465,512), with a weekly average of 155,198 tests in the entire 119-week period. Group 1 laboratories received the majority of RT-PCR tests during the first, second, and third waves (77.7%, 50.5%, and 63.8%, respectively). Table 2 shows no significant difference between the weekly average of the tests distributed in the three waves to Group 1 laboratories.

Group 2 laboratories, generally run by research groups at universities and entities that performed testing as contracted providers to SUS, represented 1.3%, 5.4%, and 11% of the total tests allocated for the first, second, and third waves (Fig 2). This corresponded to 5.2% of the total number of tests allocated, with a weekly average of 13,648 (n = 1,624,140). Student’s t-test results indicated a significant difference between the weekly average of tests distributed in the first and second wave (Table 2), which is the period this group of laboratories were provided with the highest number of tests (p < 0.001).

## Discussion

The use of RT-PCR tests in diagnosing COVID-19 has been indicated as a pandemic control measure since its onset [31]. The use of molecular tests and adequate laboratory infrastructure are essential resources in response to health emergencies, such as epidemics and pandemics, not only for surveillance but also for adequate assistance to infected people [32,33]. In this study, we analyzed the policies and resources allocated for the molecular testing of COVID-19 aimed at the epidemiological surveillance of the disease and, consequently, the control of the spread of cases and deaths.

In the first semester of 2020, the MoH announced policies to expand the diagnostic assistance network of participating laboratories, mainly by commissioning HTCs that could process significantly higher numbers of exams than those already carried out by reference laboratories in the preexisting SISLAB network. However, as the results of the distribution of tests indicate, HTCs were established in the first wave, with four units, but with a smaller number of active laboratories in the third wave, with three units in activity. Our findings confirm that an increase in the allocation of tests to the primary SISLAB network did not accompany the reduction in utilizing HTCs in the third wave. As a result, overall testing was markedly reduced in the third wave.

The public health system, the SUS, already counted on a decentralized, existing SISLAB network of public health laboratories present in all the country’s federative units and a rich experience in epidemiological surveillance. Although this network, composed mainly of LACENs and reference laboratories, was already in operation before the pandemic, the qualification of these laboratories and the allocation of a higher amount of tests only occurred in the second wave, which began in October 2020. In the second wave, the MoH managed to increase the number of laboratories and the volume of tests sent to these laboratories.

There was a reversal in the intensity of RT-PCR testing in the third wave, and the number of laboratories and the volume of tests declined. However, the decline in testing efforts contrasts with the increase in case incidence and waning immunity in the third wave. Furthermore, less than 70% of the Brazilian population had received the vaccine booster doses planned to be administered in early January 2022. Since this wave had the highest number of registered COVID-19 cases in Brazil, this decreased effort could have contributed to the exponential increase in infections.

During the pandemic, the MoH attempted to integrate and utilize veterinary, agriculture and biotechnology research-based university laboratories to expand testing in Brazil. Policies regarding public laboratories not previously associated with the MoH were limited to the beginning of the pandemic. However, these authorizations were apparently driven by public universities with a laboratory structure available, rather than a centralized federal initiative coordinated across waves and regions to increase testing coverage. Moreover, these laboratories were used mainly in the second wave. Considering the available information, verifying these laboratories’ use in epidemiological surveillance is impossible since no expansion was articulated with the MoH demand and the pandemic’s evolution. When the third wave surge began in Brazil, many public laboratories used to diagnose COVID-19 had returned to their pre-pandemic activities and did not receive RT-PCR tests.

Additionally, the distribution of tests and qualification of laboratories did not occur homogeneously among the federative units of Brazil. The inequality in public molecular laboratories certified to conduct RT-PCR testing throughout the COVID-19 pandemic across regions did not improve. Regions with more public laboratories received more tests than those with less preexisting infrastructure with the potential to contribute to molecular testing. For example, the Southeast has 53.9% of the public universities in Brazil, followed by the Northeast (21.1%), South (10.2%), North (7.6%), and Middle West (7.2%) [34].

In the case of veterinary labs, using these labs for molecular diagnostics aligns with the guidance of the World Organization for Animal Health (OIE), which developed guidelines regarding using these units to increase the COVID-19 testing effort in April 2020 [35]. The Federal Council of Veterinary Medicine [36] and the Brazilian Agricultural Research Corporation (Embrapa) [37] monitored and supported testing efforts. However, the available data do not permit us to assess the extent to which these initiatives were centrally coordinated.

In the third wave, as testing distributed to all three groups decreased, the downscaling of test distribution returned mean weekly levels to those undertaken in the first wave even though the magnitude of cases was 3.5 larger in the third wave proportionally. The number of cases registered in previous periods did not guide where the MoH sent tests in the second and third waves. Although the state of Sao Paulo has the largest resident population in the country, when looking at the number of cases registered during the pandemic, it appears that, proportionally, the pandemic was more severe in other states [38].

Although the MoH sent more than 32 million RT-PCR tests to the states by 23 November 2022 [39], and laboratories expanded equipment, staffing and hours of operation, there was a delay in the response, structural problems, lack of trained human resources, uncoordinated sample sending flow, in addition to the delay in processing, recording, and delivery of results [40].

Public health scientists concur that RT- PCR tests remained an essential tool for pandemic control due to their greater sensitivity and, mainly, to the link between public laboratories and the MoH surveillance system. These tests, however, are costly and require significant processing time. Rapid antigen tests have advantages concerning quick access to test results and lower cost, but they vary in sensitivity. As antigen tests began to be used in more significant volumes in the public health system from 2021 onwards, molecular testing efforts and genomic surveillance were reduced in Brazil [41]. Thus, rather than being used to expand testing, antigen tests resulted in a decreased diagnostic effort in public molecular laboratories, thus weakening the MoH’s efforts to monitor and better control the pandemic. Furthermore, the public health system’s ability to ensure surveillance and monitoring across the country also occurred in a manner that magnified regional inequalities. The findings reported in this study underscore that centralized and fast coordination is essential to activate preexisting resources and infrastructure during an emergency and to ensure that systems are continually strengthened throughout a prolonged crisis, such as COVID-19.

This study has several limitations. Our study is based on the number of tests distributed by the MoH. Although the laboratories were geographically spread across the country, we were not able to assess if tests were distributed to minimize logistic delays. Due to the lack of data on RT-PCR test processing, we are unable to verify the extent to which the allocation of more significant supplies in specific periods and regions resulted in a higher amount of processed tests in recipient labs. Furthermore, future research should also analyze the federal government’s investments to support the expansion of production capacity in public clinical laboratories by examining investments in equipment (e.g., automated test processing) and qualified personnel (e.g., data and lab technicians). State and local governments also invested efforts to expand molecular diagnosis of COVID-19, but these programs were not analyzed. Future research should explore the extent to which federal policies complemented and ensured further equity across the Brazilian federation, considering these local initiatives.

## Conclusion

Surveillance efforts are vital to prevention efforts and interventions to control disease during health emergencies. Countries have expanded their molecular diagnostic laboratory infrastructure to control infectious diseases in epidemic scenarios, even in resource-limited settings [42]. However, expanding testing and enhancing laboratory-based surveillance requires resources, training and careful planning [43]. This article has documented considerable challenges that affected the implementation of an effective and sustained molecular testing program for SARS-CoV-2 in the public health system due to the need to coordinate efforts across public health, clinical and research laboratories during the first three major pandemic waves in Brazil.

Similar to other countries, Brazil appeared to have the nominal capacity to rapidly develop, implement and expand PCR-based test assays using its distribution network and an array of public health laboratories. However, as the country experienced some of the highest infection, mortality, and excess mortality rates due to the COVID-19 pandemic and experienced the unexpected collapse of public health services as the epidemic accelerated across the country, molecular testing struggled to meet ever-increasing public health clinical diagnostics and surveillance needs. During an extended emergency, such as the first three waves of COVID-19 outbreak, there are also major challenges in ensuring sustained levels of molecular testing.

## Supporting information

S1 Fig Pearson’s correlation for weekly average of RT-PCR tests distributed during the COVID-19 second wave (x-axis) and the number of COVID-19 cases (per 100,000 inhabitants) during the first wave in Brazil (y-axis). (TIF)

S2 Fig Pearson’s correlation for weekly average of RT-PCR tests distributed during the COVID-19 second wave (x-axis) and the number of COVID-19 deaths (per 100,000 inhabitants) during the first wave in Brazil (y-axis). (TIF)

S3 Fig Pearson’s correlation for weekly average of RT-PCR tests distributed during the COVID-19 third wave (x-axis) and the number of COVID-19 cases (per 100,000 inhabitants) during the second wave in Brazil (y-axis). (TIF)

S4 Fig Pearson’s correlation for weekly average of RT-PCR tests distributed during the COVID-19 third wave (x-axis) and the number of COVID-19 deaths (per 100,000 inhabitants) during the second wave in Brazil (y-axis). (TIF)

S1 Table COVID-19 Diagnostic criteria for testing and official confirmation of cases issued by the Ministry of Health, 2020–2022. (DOCX)
