# Supplementary figures and images for: An assessment of the public health surveillance strategy based on molecular testing during three major pandemic waves of COVID-19 in Brazil
Source: PLOS Glob Public Health. 2023 Aug 18;3(8):e0002164. doi: 10.1371/journal.pgph.0002164 (PMC10437824; doi:10.1371/journal.pgph.0002164)

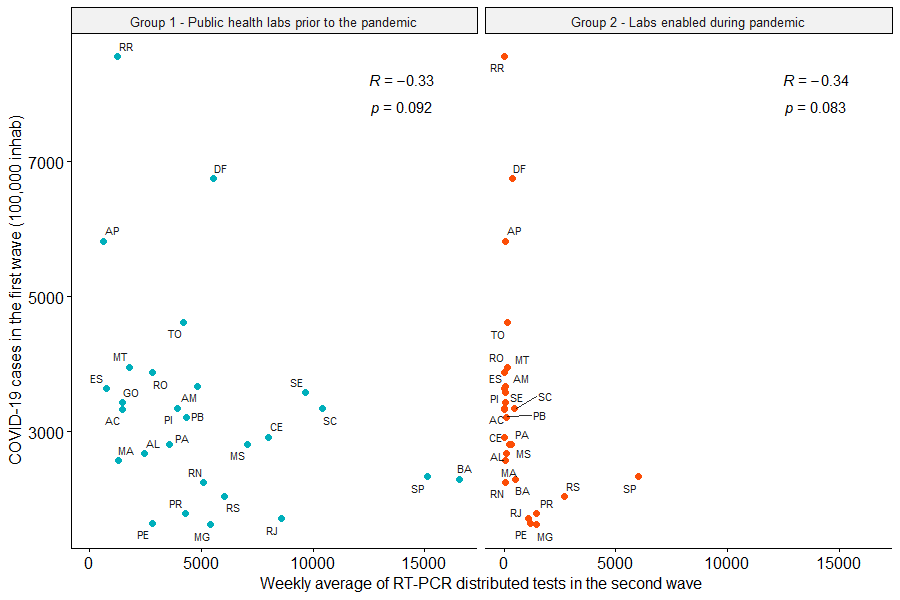

Supplement: S1 Fig — (TIF) [file pgph.0002164.s001.tif]

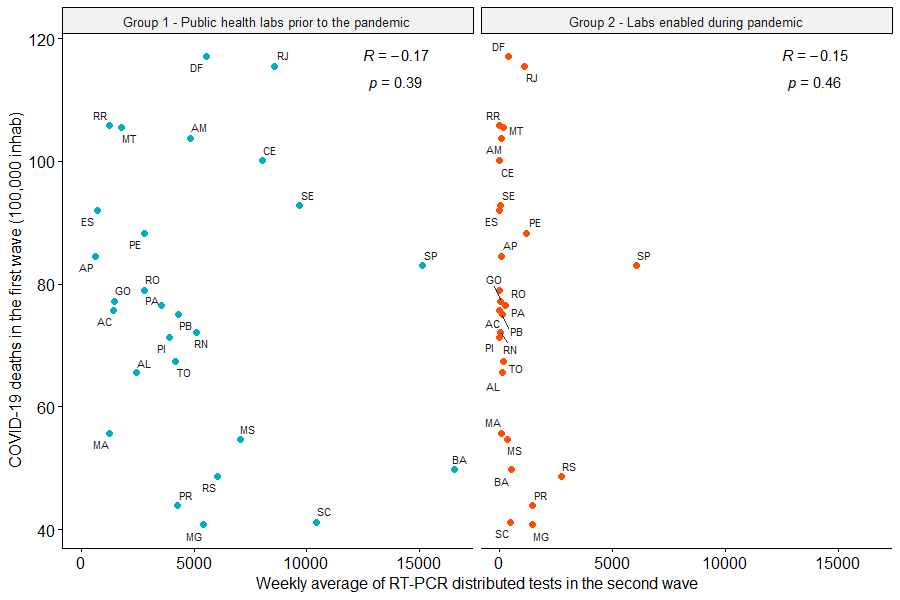

Supplement: S2 Fig — (TIF) [file pgph.0002164.s002.tif]

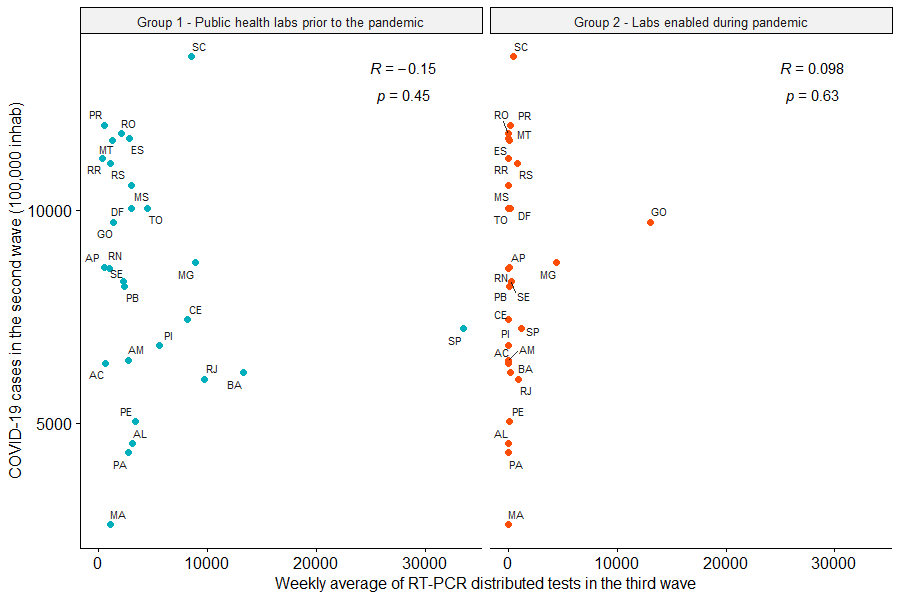

Supplement: S3 Fig — (TIF) [file pgph.0002164.s003.tif]

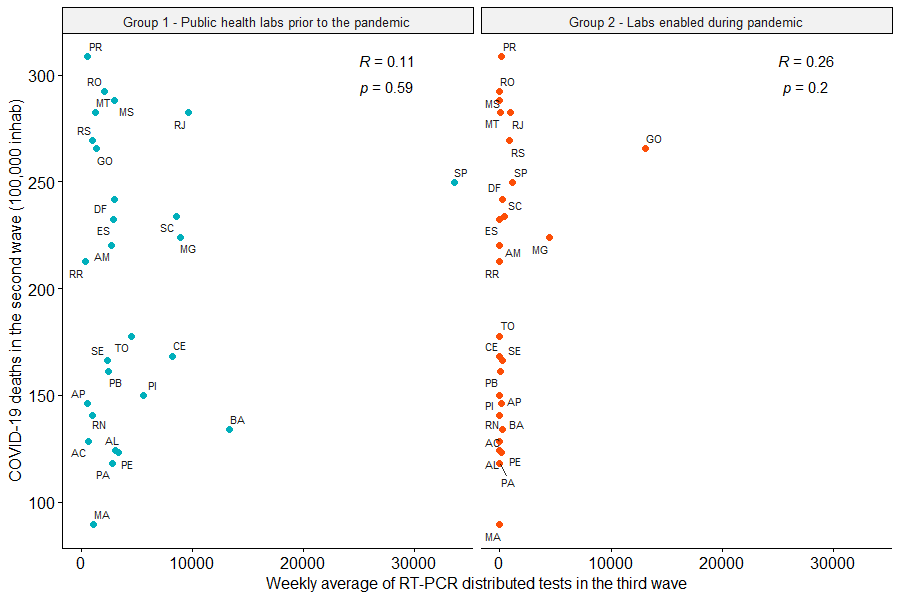

Supplement: S4 Fig — (TIF) [file pgph.0002164.s004.tif]
