# Supplementary material for: An assessment of the public health surveillance strategy based on molecular testing during three major pandemic waves of COVID-19 in Brazil
Source: PLOS Glob Public Health. 2023 Aug 18;3(8):e0002164. doi: 10.1371/journal.pgph.0002164 (PMC10437824; doi:10.1371/journal.pgph.0002164)
Supplement: S1 Table — (DOCX) [file pgph.0002164.s005.docx]

| **Table S1. COVID-19 Diagnostic Criteria for Testing and Official Confirmation of Cases issued by the MoH, 2020-2022** | | | | | |
| --- | --- | --- | --- | --- | --- |
|  | Bulletin3 (21 February 2020) | Guidance, Version 1.0  (6 April 2020) | Guidance, Version 2.0  (5 August 2020) | Guidance, Version 3.0  (15 March 2021) | Guidance, Version 4.0  (May 2022) |
| RT-PCR Tests | Yes | Yes | Yes | Yes | Yes |
| RT-PCR Lamp Tests | No | No | Yes | Yes | Yes |
| Serology Tests | No | Yes  (IgM and/or IgG) | Yes  (IgM and/or IgG) | Only for individuals who are not vaccinated.  (IgM, IgA and/or IgG*) | Only for individuals who are not vaccinated.  (IgM, IgA and/or IgG*) |
| Antigen Tests | No | No | Yes | Yes | Yes |
| Clinical-Epidemiological Criteria | No | Yes | Yes | Yes | Yes |
| Clinical Criteria | No | No | Yes | Yes | Yes |
| Clinical and Imaging Criteria | No | No | Yes | Yes | Yes |
| Hospitalized Case | No | Yes | Yes | Yes | Yes |
| Guidance on contacts of a positive case |  | No | Contact tracing should be used for all confirmed cases. | Contact tracing should be used for all confirmed cases. | Contact tracing should be used for all confirmed cases. |
| Guidance on symptomatic testing |  | No | Yes | Yes | Yes |
| Guidance on Asymptomatic Testing |  | No | Yes | Yes | Yes |
| General Guidance on Notification |  | SARI and Flu-like cases, SARI hospitalized or deaths regardless of hospitalization, which  meets the case definition.” | Flu-like cases, SARI hospitalized and deaths regardless of hospitalization, which meets the case definition. Asymptomatic individuals with laboratory confirmation by molecular biology or immunology of recent COVID-19 infection. | Flu-like cases, SARI hospitalized and deaths regardless of hospitalization, which meets the case definition. Asymptomatic individuals with laboratory confirmation by molecular biology or immunology of recent COVID-19 infection. | Flu-like cases, SARI hospitalized and deaths regardless of hospitalization, which meets the case definition. Asymptomatic individuals with laboratory confirmation by molecular biology, antigen testing, or immunological testing that show recent COVID-19 infection. |

Sources: BRASIL. Ministério da Saúde. Secretaria de Vigilância em Saúde. Guia de Vigilância Epidemiológica. Emergência de Saúde Pública de Importância Nacional pela Doença pelo Coronavírus 2019. Brasília, 2020-2022.

*The guide explains in Portuguese, “*Considerando a história natural da covid-19 no Brasil, um resultado isolado de IgG reagente No deve ser considerado como teste confirmatório para efeitos de notificação e confirmação de caso. Um resultado IgG reagente deve ser usado como critério laboratorial confirmatório somente em indivíduos No vacinados, sem diagnóstico laboratorial anterior para covid-19 e que tenham apresentado sinais e sintomas compatíveis, com no mínimo 8 dias antes da realização desse exame*”.
